# Supplementary material for: Exploring and prioritising strategies for improving uptake of postnatal care services in Thyolo, Malawi: A qualitative study
Source: PLOS Glob Public Health. 2024 Mar 6;4(3):e0002992. doi: 10.1371/journal.pgph.0002992 (PMC10917263; doi:10.1371/journal.pgph.0002992)
Supplement: S1 Text — (DOCX) [file pgph.0002992.s001.docx]

**Supplementary File 1- Focus Group Discussion Guide**

**Perceptions on PNC Services:**

1. What is your understanding of postnatal care services?

Probe on:

- What a mother and baby should get and receive as part of PNC
- Beliefs on PNC- individual and communities
- PNC time points for Mother and Newborn

1. What are your roles in PNC services for women and newborns in the community?

**Barriers to PNC**

1. Explain to me in details the factors that impede PNC services utilization by mothers and newborns in this community?

Probe on:

- Cultural and religious beliefs
- Health System Factors
- Condition of patient
- Community norms
- Orphaned Neonates
- Mothers whose baby died

**Enablers to PNC Services**

1. Explain to me the factors that enable mothers and newborns to receive PNC services in this community?

Probe on

- Culture
- Health system factors
- Community Factors
- Individual factors
- Occupation – including farming
- Orphaned Neonates
- Mothers whose baby died

**Strategies for improving provision and Uptake of PNC services**

1. Explain to me the strategies that can be employed to improve provision of PNC services to mothers and newborns in this community?

Probe on:

- Patient Centred Approaches
- Community based approaches
- Facility Based approaches

**NB- For postnatal women FGD- inquire on the PNC services they received, when, where and whom?**

**Closing Remarks**

1. We are now at the end of the discussion, is there anything that you would like to add concerning provision and uptake of PNC services among mothers and newborns?
