# Supplementary material for: Exploring and prioritising strategies for improving uptake of postnatal care services in Thyolo, Malawi: A qualitative study
Source: PLOS Glob Public Health. 2024 Mar 6;4(3):e0002992. doi: 10.1371/journal.pgph.0002992 (PMC10917263; doi:10.1371/journal.pgph.0002992)
Supplement: S2 Text — (DOCX) [file pgph.0002992.s002.docx]

**Supplementary File 2- In-depth Interview Guide**

**Section A -Welcome Remarks**

1. Tell me about the common maternal and newborn illnesses in this area.
2. What is your role at this facility?
3. What is your role in PNC Services?

**Section B - Perceptions on PNC Services:**

1. What is your understanding of postnatal care services?

Probe on:

- Components of PNC for Mother and Newborn
- PNC time points for Mother and Newborn

**Section C- Barriers to PNC**

1. Explain to me in details the factors that impede provision of PNC services at this facility?

Probe on:

- Resources
- Volume of Work
- Training
- Capabilities and Knowledge
- Culture
- Operation hours
- Condition of patient
- Operating procedures such as discharge times

1. Explain to me the patient factors that impede uptake of PNC services in this area?

Probe on:

- Cultural Beliefs
- Condition of patient
- Community Norms
- Socioeconomic Status
- Education Status
- Occupation – including farming
- Orphaned Neonates
- Mothers whose baby died

**Section D- Enablers to PNC Services**

1. Explain to me the factors that enable health care workers to provide PNC services at this facility

Probe on

- Resources
- Volume of Work
- Training
- Capabilities and Knowledge
- Culture
- Operation hours
- Condition of patient
- Operating procedures such as discharge times

1. Explain to me the factors that enable Postnatal mothers and newborns to take up PNC services at this facility

Probe on:

- Cultural Beliefs
- Condition of patient
- Community Norms
- Socioeconomic Status
- Education Status
- Occupation – including farming
- Orphaned Neonates
- Mothers whose baby died

**Section E- Strategies for improving provision and Uptake of PNC services**

1. Explain to me the strategies that can be employed to improve provision of PNC services at this facility?

Probe on:

- Task Shifting Approaches
- Patient Centred Approaches
- Community based approaches
- Facility Based approaches

1. Explain to me the strategies that can be employed to improve uptake of PNC services by Mothers and Newborns at this facility?

Probe on:

- Task Shifting Approaches
- Patient Centred Approaches
- Community based approaches
- Facility Based approaches

**Section F- Closing Remarks**

1. We are now at the end of the Interview, Is there anything that you would like to add concerning provision and uptake of PNC services among mothers and newborns?

Thank you very much for your time.
