# Supplementary material for: Exploring and prioritising strategies for improving uptake of postnatal care services in Thyolo, Malawi: A qualitative study
Source: PLOS Glob Public Health. 2024 Mar 6;4(3):e0002992. doi: 10.1371/journal.pgph.0002992 (PMC10917263; doi:10.1371/journal.pgph.0002992)
Supplement: S3 Text — (DOCX) [file pgph.0002992.s003.docx]

**Supplementary File 3-Demographic Characteristics of Postnatal Women, Men, and Elderly Women N=72**

| **Variable** | **PNC women n=24** | **Men n=24** | **Elderly Women n=24** |
| --- | --- | --- | --- |
| Age range | 18-36 | 28-72 | 42-73 |
| Employment  Housewives  Businesses  Farmers  Employed | 9  7  5  3 | 0  14  7  3 | 0  6  18  0 |
| Education  None  Primary  Secondary  College | 0  9  13  2 | 0  16  8  0 | 3  21  0  0 |
