# Supplementary material for: Exploring and prioritising strategies for improving uptake of postnatal care services in Thyolo, Malawi: A qualitative study
Source: PLOS Glob Public Health. 2024 Mar 6;4(3):e0002992. doi: 10.1371/journal.pgph.0002992 (PMC10917263; doi:10.1371/journal.pgph.0002992)
Supplement: Attachment — Submitted filename: Review comments for PLOSONE.docx [file pgph.0002992.s004.docx]

|  | **Comments** |
| --- | --- |
| **Tittle** | The tittle “Exploring and prioritising strategies for improving uptake of postnatal care services in  Thyolo, Malawi: a qualitative study” is interesting and relevant. |
| **Abstract** | The introduction and results of the study seem to vary slightly. While the introduction sought to explore and prioritized the strategies for optimizing the uptake of postnatal care (PNC), the results settle on classified strategies (Thus demand and supply). My opinion is that the demand and supply need to be mentioned in introduction as well, otherwise, it looks strange to get these terms on results section. Apart from these, the authors seem to answer the research question. |
|  |  |
| **Introduction and background** | The author indicated that maternal (30%) and neonatal deaths (17 per 1000 live births) are high in postnatal period globally, however does not indicate if this is Malawi problem also. Secondly, I feel there is need to highlight contributing factors/causes to reported maternal deaths. This may provide background to development of relevant strategies. The study does not state if some of these deaths occurred due to TB.  The concluding paragraph points out the relevance of PNC to backup need for a study to identify contextual strategies that optimise the uptake of PNC care in Malawi. Nonetheless, this has not been tackled clearly in earlier paragraphs. I feel, the preceding paragraph needed to provide how provide this, at least for elsewhere. |
|  |  |
| **Methods** | There are enough data to draw a conclusion |
|  | Authors address possible limitations of the research |
|  | Data were collected and interpreted accurately. Exceptional are the following areas: |
|  | On recruitment (Page 9): Fathers were identified from their own residences. How did the researcher conduct FGD of this group, a little more clarity may be needed. |
|  |  |
|  | On data collection (Page 9): Can the authors indicate where and on which population did they test the topic and the study guide? Is it on the same population or another? |
|  |  |
| **Results** | Good results, Seem to support conclusions. |
|  |  |
|  | However, need to: |
|  | Highlight the variations in responses among FGD groups in all the study sites. |
|  | As stated earlier, grouping results to demand side strategies and supply side strategies seem to be new phenomena just introduced. There is need to introduce these terms to the reader in the introduction. |
|  |  |
| **Statistical analysis** | The data provide enough evidence for the authors’ conclusions. |
|  | I feel the authors have provided a sufficient amount of data and information for other researchers to recreate the analyses |
| **Conclusions** |  |
