# Supplementary material for: Exploring and prioritising strategies for improving uptake of postnatal care services in Thyolo, Malawi: A qualitative study
Source: PLOS Glob Public Health. 2024 Mar 6;4(3):e0002992. doi: 10.1371/journal.pgph.0002992 (PMC10917263; doi:10.1371/journal.pgph.0002992)
Supplement: Attachment — Submitted filename: Letter of Response.docx [file pgph.0002992.s005.docx]

Dear Editor and Reviewers,

Thank you for the constructive review of our manuscript which has been beneficial to us. Please find below the responses to the queries raised by the reviewers.

**Reviewers' comments:**

**Reviewer #1:**

Congratulations to the authors for the great effort in putting this paper together. Some few grammatical errors regarding punctuations noted across the work. I recommend minor revisions to the work prior to publication. Please consider the comments below for revision.

**Response: Thank you for this comment and the constructive review.**

ABSTRACT:
1. Your abstract is too long, standard word limit should not exceed 250 words. Please consider editing the abstract to not more than 250 word count.

**Response: The abstract has been shortened to below 250 words.**
.

2. Insert a "full stop" at the end of the first sentence under methods in the abstract.

Response: A full stop is now in place.

KEY WORDS:
Consider deleting the "coma" punctuation at the end of the last key word.
Response: Thank you for this suggestion, punctuation at the end has been revised

**INTRODUCTION:**

1. Paragraph 1, sentence 3, intext citation appears as superscript inappropriately written

**Response: Thank you for noting this. This has been corrected.**

2. Paragraph 2, 2nd but last sentence, normal temperature ranges between 36.5 to 37.5 and not 35.5

**Response: Thanks for noting this misrepresentation of the literature. It has been revised to reflect that there is a need to assess for a low body temperature and it now reads as follows:**

**a low body temperature lower than 35.5** **degrees Celsius.**

**This is on page 4.**

3. Consider inserting "and" before hyperbilirubinemia in the last sentence

**Response: This has been inserted, thank you.**

4. Paragraph 4, Average days should be approximated to hours and not decimals. For example, 0.5days could be written as 12hours

**Response: This has been revised to reflect half a day to 9 days. We refrained from calculating hours because we felt that the days are easier to communicate in practice as opposed to hours especially when they extend over 72 hours.**

5. Paragraph 5, percentage of PNC visits of mothers and newborns within 48hours (84% and 88% respectively) contradicts with the less than 50% PNC attendance reported in the abstract of the manuscript. Secondly, these percentages does not depict a serious health challenge that requires investigation. Which is which?

**Response: The abstract has been updated and no longer has the contradicting information. We agree with you that immediate PNC is high and we have further shown how the rates fall in the successive visits at one week and six weeks hence the need to optimize it.**

**Please note that the 50% rate was from the last DHS in 2015 which has been superseded with the Multiple Indicator Cluster Survey results of 2019- 2020.**

METHODOLOGY:
A. Design: Delete punctuation mark "coma" before (HSA) in sentence 4.

**Response: We have deleted the coma.**

B. Setting: Correct the grammatical error and insert punctuation mark appropriately in the last sentence

**Response: The last sentence has been revised and now reads as follows:**

**Unlike the other facilities, which primarily employ nurse-midwife technicians and medical assistants, Facility 3 has a diverse range of healthcare professionals who provide maternity services. These professionals include nursing officers, clinical officers, and medical officers, who have training at the bachelor's and master's levels.**

**This is on page 7.**

C. Sampling and Sample size: More information is required in this section
1. What was the total sample size for the study and how was the total sample size determined for IDIs and FGDs? Please include that in the writeup.

**Response- We have added the following information
Our goal was to have a sample that would enable us to obtain the rich data required for every technique of data collection. We performed seven in-depth interviews in total, with the midwives who provided care in the facilities and the nursing and midwifery in charges of the maternity wards in all three facilities. Saturation of ideas as well as information power by ensuring selection of participants with a depth of information in PNC services, specifying the roles they play, and the data collection approach of engaging them in the dialogue guided the data collection approach to achieving an adequate sample size. In the focus group discussions, we settled for a focus group discussion per site per group of people and held a total of 12 focus group discussions, translating into four FGDs per site. We believed that with 12 FGDs, we would reach 90% of the themes as suggested by Guest et al.**

**This is on page 7.**

2. Please be consistent with the numbering system, either write in numerals or in words. For example use either 1, 2, 3 or one, two, three

**Response: Please guide, the rule of thumb has been to spell out numbers less than 10 and only write them in numerical if they are above 10. Please Editor, provide guidance if this is not the same with PGPH.**

3. What were the inclusion and exclusion criteria for selecting participants?

**Response: We have included the eligibility criteria as follows:**

**We included healthcare workers that were willing to participate in the study, work in maternity, postnatal, or as HSAs, and were 18 years of age and older.**

**We excluded students from various institutions and those who had worked for less than a month in postnatal care settings. We included men in FGDs who were available and willing to participate, those with a baby who was less than a year old, as determined by asking for the child’s health passport book. We included elderly women who were responsible for counseling women on maternity aspects, recognised as resource persons for maternity aspects in a community, and grandmothers of grandchildren under 12 months of age. We included postnatal women who were willing to participate in the study, postnatal women with or without a living baby. If with a living baby, the age of the baby was varied: immediately after birth, 24 hours, 48 hours, 7 days, and 42 days after birth; we excluded postnatal women that were physically and mentally unstable mothers.**

**The additions are on page 8.**

RESULTS: Additional information is required in this section.
1. How many themes and subthemes emerged from the analysis? This could be stated or tabulated for understanding.

**Response: There were three main subthemes that were realised in this study and these were:**

**Demand-Side Strategies- This theme had four subthemes namely appointment date reminders, provision of free health passport books, community awareness campaigns, and involvement of men in the services.**

**Supply Side Strategies – This theme had four subthemes namely training health providers having infrastructure for the services, linkage to other services and improving clinic operations. Improving clinic operations had two categories namely task-shifting and hours of operation.**

**Cross-cutting strategies**

**The cross-cutting strategy that was suggested was having clinics closer to users’ areas of residence. This was raised on the demand as well as supply side.**

**To ease with the visualization of the themes and subthemes, we have included a Figure to illustrate the themes.**

Demand Side Strategies: appointment date reminders, provision of free health passport books, community awareness campaigns, and involvement of men in the services

Crosscutting Strategy: Proximity of clinics to areas of residence

Supply Side Strategies: training health providers having infrastructure for the services, linkage to other services and improving clinic operations (task-shifting and hours of operation)

Figure 1: Summary of themes and subthemes of the proposed strategies

2. Be consistent in writing participant IDs after each quotation. For example you could chose to stick to the format of (Facility, category of participant, and means of data collection).

**Response: Thank you for this comment, we have reviewed and are now consistent in the identification of the participants.**

DISCUSSION:
1. Consider inserting a "full stop" at the end of the first sentence

**Response: Thank you for this comment and we have added a full stop.**

2. Inappropriate font use, consider editing font type and size in the 5th paragraph (3rd but last sentence)

**Response: We appreciate for noting this and the font size has been revised to be consistent in the document.**

CONCLUSION:
Consider inserting a "full stop" at the end of the first sentence

**Response: Thank you for this and a full stop is inserted.**

Thank you

**Response: We appreciate the thorough review to our manuscript.**

**Reviewer #2:**

The manuscript is technically sound, and the data support the conclusions. It provides a description that is methodologically and ethically rigorous. No statistical analysis is applicable because the approach is qualitative.

**Response: Thank you very much for this comment.**

**Reviewer #3:**

Title: The title “Exploring and prioritising strategies for improving uptake of postnatal care services in Thyolo, Malawi: a qualitative study” is interesting and relevant.

**Response: Thank you for this comment.**

Abstract: The introduction and results of the study seem to vary slightly. While the introduction sought to explore and prioritized the strategies for optimizing the uptake of postnatal care (PNC), the results settle on classified strategies (Thus demand and supply). My opinion is that the demand and supply need to be mentioned in introduction as well, otherwise, it looks strange to get these terms on results section. Apart from these, the authors seem to answer the research question.

**Response: We have taken this into consideration and have clarified about the strategies being on the demand or supply spectrum in the introduction. Refer to page 5.**

**The strategies can be on the demand side as per the end-user’s preference or can be on the supply side which includes those that stem from the health facility. The demand strategies in place for optimizing uptake of PNC services include community awareness of the importance of PNC [7], offering PNC services in the communities where women reside [8, 9]. The supply -side strategies include task shifting of services to community-based health workers [9, 10], and training of HCWs on the required assessments to be delivered [9].**

Introduction and background

The author indicated that maternal (30%) and neonatal deaths (17 per 1000 live births) are high in postnatal period globally, however does not indicate if this is Malawi problem also.

**Response: We have included the following information to show the burden in Malawi. The new sentence reads as follows on page 3:**

**In Malawi, 65% of maternal deaths occur in the postpartum period while most infants die in the neonatal period with a neonatal mortality rate of 26 deaths per 1000 livebirths whereas the post neonatal mortality rate is at 14 deaths per 1000 livebirths.**

Secondly, I feel there is need to highlight contributing factors/causes to reported maternal deaths. This may provide background to development of relevant strategies. The study does not state if some of these deaths occurred due to TB.

**Response:** **According to the recent Maternal Death Report, maternal sepsis (25%), eclampsia (20%), hemorrhage (13%) are the main causes of maternal deaths in Malawi. Inadequate monitoring of a woman in labor, delays in acting and exposing a woman to prolonged abnormal observations, and inadequate emergency skills are additional factors related to health workers. Additional factors include delays in receiving health care, communication and transportation difficulties, and supply shortages.**

**This is on page 3.**

The concluding paragraph points out the relevance of PNC to backup need for a study to identify contextual strategies that optimise the uptake of PNC care in Malawi. Nonetheless, this has not been tackled clearly in earlier paragraphs. I feel, the preceding paragraph needed to provide how provide this, at least for elsewhere.

**Response: The beginning of the same paragraph alludes to the strategies that are available elsewhere.**

Methods

There are enough data to draw a conclusion. Authors address possible limitations of the research. Data were collected and interpreted accurately.

**Response: Thank you for these comments.**

Data were collected and interpreted accurately. Exceptional are the following areas .

On recruitment (Page 9): Fathers were identified from their own residences. How did the researcher conduct FGD of this group, a little more clarity may be needed.

**Response: We have included the following:** **Upon identification of eligible men for an FGD, they were booked to attend a discussion on a specific day and were asked to report to the facility for the FGD.**

**Refer to page 10.**

On data collection (Page 9): Can the authors indicate where and on which population did they test the topic and the study guide? Is it on the same population or another?

**Response: We have revised the section on pretesting, and it now reads as follows:**

**Before data collection, the topic and interview guides were tested on a comparable population that was found at Facility 1 to ascertain their ability to gather the information that was needed. The findings from the pilot phase were not included in the overall results however they highlighted areas that needed revisions to allow for more probing.**

**Refer to page 11.**

Results: Good results, Seem to support conclusions

**Response: Thank you for this comment**

However, need to Highlight the variations in responses among FGD groups in all the study sites.

**Response: The responses were similar and had no variation hence we did not indicate that on that basis.**

As stated earlier, grouping results to demand side strategies and supply side strategies seem to be new phenomena just introduced. There is need to introduce these terms to the reader in the introduction.

**Response: These terms are now introduced in the introduction section.**

Statistical Analysis and Conclusions

The data provide enough evidence for the authors’ conclusions. I feel the authors have provided enough data and information for other researchers to recreate the analyses.

**Response: Thank you for this comment.**

Sincerely,

Alinane Linda Nyondo-Mipando (Corresponding Author)
